# Supplementary material for: Tunable elliptical cylinders for rotational mechanical studies of single DNA molecules
Source: Sci Adv. 2024 Dec 13;10(50):eadr4519. doi: 10.1126/sciadv.adr4519 (PMC11641005; doi:10.1126/sciadv.adr4519)
Supplement: Supplementary file 1 — Supplementary Text Figs. S1 to S7 References [file sciadv.adr4519_sm.pdf]

Science Advances
AAAS

Supplementary Materials for

Tunable elliptical cylinders for rotational mechanical studies of single DNA molecules

Yifeng Hong *et al.*

Corresponding author: Michelle D. Wang, mwang@physics.cornell.edu

Sci. Adv. **10**, eadr4519 (2024)
DOI: 10.1126/sciadv.adr4519

This PDF file includes:

Supplementary Text
Figs. S1 to S7
References

Cylinders Tilting in the Trapping Beam

The AOT measures the torque on the cylinder about its cylindrical axis, so rotation about the other two orthogonal axes is undesirable. The elongated cylindrical shape is chosen to ensure that a trapped cylinder aligns its cylindrical axis with the direction of the trapping beam propagation. To limit cylinder rotation around the other two axes, the cylinder must have a high angular trapping stiffness around those axes to resist tilting(*19*).

To evaluate the angular trapping stiffness around those two axes, we performed COMSOL simulations using the relative permittivity tensors of the metamaterial elliptical cylinder:

$$\overleftrightarrow{\epsilon}_{r,\theta_x} = \begin{bmatrix} n_{\mathrm{o}}^2 & 0 & 0 \\ 0 & n_{\mathrm{e}}^2\sin^2\theta_x + n_{\mathrm{o}}^2\cos^2\theta_x & (-n_{\mathrm{e}}^2 + n_{\mathrm{o}}^2)\sin\theta_x\cos\theta_x \\ 0 & (-n_{\mathrm{e}}^2 + n_{\mathrm{o}}^2)\sin\theta_x\cos\theta_x & n_{\mathrm{e}}^2\cos^2\theta_x + n_{\mathrm{o}}^2\sin^2\theta_x \end{bmatrix}, \quad \text{(S1)}$$

$$\overleftrightarrow{\epsilon}_{r,\theta_y} = \begin{bmatrix} n_{\mathrm{e}}^2\sin^2\theta_y + n_{\mathrm{o}}^2\cos^2\theta_y & 0 & (n_{\mathrm{e}}^2 - n_{\mathrm{o}}^2)\sin\theta_y\cos\theta_y \\ 0 & n_{\mathrm{o}}^2 & 0 \\ (n_{\mathrm{e}}^2 - n_{\mathrm{o}}^2)\sin\theta_y\cos\theta_y & 0 & n_{\mathrm{e}}^2\cos^2\theta_y + n_{\mathrm{o}}^2\sin^2\theta_y \end{bmatrix}, \quad \text{(S2)}$$

where θ_x and θ_y are the tilting angles around the elliptical cylinder's major axis and minor axis, respectively. Compared to an isotropic elliptical cylinder, a metamaterial elliptical cylinder can enhance the angular stiffness about its minor axis by 3-fold (Fig. S2), resulting in more robust anti-tilting around this axis.

We performed similar simulations for a quartz cylinder, which has the following relative permittivity tensors:

$$\overleftrightarrow{\epsilon}_{r,\theta_x} = \begin{bmatrix} n_{\mathrm{e}}^2 & 0 & 0 \\ 0 & n_{\mathrm{o}}^2 & 0 \\ 0 & 0 & n_{\mathrm{o}}^2 \end{bmatrix}, \quad \text{(S3)}$$

$$\overleftrightarrow{\epsilon}_{r,\theta_y} = \begin{bmatrix} n_{\mathrm{e}}^2\cos^2\theta_y + n_{\mathrm{o}}^2\sin^2\theta_y & 0 & (-n_{\mathrm{e}}^2 + n_{\mathrm{o}}^2)\sin\theta_y\cos\theta_y \\ 0 & n_{\mathrm{o}}^2 & 0 \\ (-n_{\mathrm{e}}^2 + n_{\mathrm{o}}^2)\sin\theta_y\cos\theta_y & 0 & n_{\mathrm{e}}^2\sin^2\theta_y + n_{\mathrm{o}}^2\cos^2\theta_y \end{bmatrix}, \quad \text{(S4)}$$

where the permittivity tensor is independent of the tilting angle θ_x around the cylinder's extraordinary axis, and θ_y is the tilting angle around the ordinary axis. When having the same volume, the metamaterial elliptical cylinder is about 2~4-fold more resistant to tilting than the quartz cylinder around its extraordinary axis and ordinary axis (Fig. S2).

Detailed Fabrication Protocol of Metamaterial Elliptical Cylinders

1. Clean the Si wafer (Ultrasil, Lot# 4-14359) with a hot piranha solution.
2. Deposit ~ 100 nm Al_2O_3 sacrificial layer onto the Si wafer via evaporation.
3. Deposit Si_3N_4 then SiO_2 with a single layer thickness of ~ 60 nm through plasma-enhanced chemical vapor deposition (PECVD).
4. Repeat step 3 for 5 times, resulting in a total thickness of ~ 600 nm metamaterial.
5. Activate the surface with 20 min O_2 plasma.
6. React with (3-Aminopropyl)triethoxysilane solution(*19*).
7. Coat the anti-reflection coating DS-K101, then soft bake at 185 °C for 90 s.

8. Coat the photoresist UV-210-0.6, then soft bake at 135 °C for 90 s.
9. DUV lithography with a photomask of ellipses (eccentricity = 0.6), followed by a post-exposure bake at 135 °C for 90 s.
10. Develop in AZ 726 MIF developer for 60 s.
11. Hard bake at 115 °C for 60 s.
12. O_2 descum for 30 s.
13. Dry etch (reactive ion etching) with the chemistry of 45 sccm CHF_3, 15 sccm Ar, 50 mTorr, 200 W until reaching the Al_2O_3 layer.
14. Remove the photoresist with heated Microposit Remover 1165 along with sonication.
15. Liftoff the metamaterial elliptical cylinders with AZ 726 MIF developer for ~ 3 hr.
16. Collect the cylinder through a centrifuge.

Detailed Fabrication Protocol of Isotropic SiO_2 Elliptical Cylinders

We also fabricated isotropic SiO_2 elliptical cylinders to verify the methodology of using shape anisotropy for torque generation on the AOT. The fabrication protocol of those cylinders is listed as follows:

1. Clean the Si wafer (Ultrasil, Lot# 4-14359) with a hot piranha solution.
2. Deposit ~ 100 nm Al_2O_3 sacrificial layer onto the Si wafer via evaporation.
3. Deposit ~ 1 µm SiO_2 through PECVD.
4. Activate the surface with 20 min O_2 plasma.
5. React with (3-Aminopropyl)triethoxysilane solution(*19*).
6. Coat the anti-reflection coating DS-K101, then soft bake at 185 °C for 90 s.
7. Coat the photoresist UV1400-1.4, then soft bake at 135 °C for 90 s.
8. DUV lithography with a photomask of ellipses (eccentricity = 0.6), followed by a post-exposure bake at 115 °C for 90 s.
9. Develop in AZ 726 MIF developer for 60 s.
10. Hard bake at 110 °C for 60 s.
11. O_2 descum for 70 s.
12. Dry etch (reactive ion etching) with the chemistry of 45 sccm CHF_3, 15 sccm Ar, 50 mTorr, 200 W until reaching the Al_2O_3 layer.
13. Remove the photoresist with heated Microposit Remover 1165 along with sonication.
14. Liftoff the metamaterial elliptical cylinders with AZ 726 MIF developer for ~ 3 hr.
15. Collect the cylinder through a centrifuge.

Plasmid (pMDW133) Sequence

gatcctctagagtcgacctgcaggcatgcaagcttggcgtaatcatggtcatagctgtttcctgtgtgaaattgttatccgctcacaattccacacaacatacgagccggaagcataaagtgtaa
agcctggggtgcctaatgagtgagctaactcacattaattgcgttgcgctcactgcccgctttccagtcgggaaacctgtcgtgccagctgcattaatgaatcggccaacgcgcggggagag
gcggtttgcgtattgggcgctcttccgcttcctcgctcactgactcgctgcgctcggtcgttcggctgcggcgagcggtatcagctcactcaaaggcggtaatacggttatccacagaatcagg
ggataacgcaggaaagaacatgtgagcaaaaggccagcaaaaggccaggaaccgtaaaaaggccgcgttgctggcgtttttccataggctccgcccccctgacgagcatcacaaaaatc
gacgctcaagtcagaggtggcgaaacccgacaggactataaagataccaggcgtttccccctggaagctccctcgtgcgctctcctgttccgaccctgccgcttaccggatacctgtccgcc
tttctcccttcgggaagcgtggcgctttctcatagctcacgctgtaggtatctcagttcggtgtaggtcgttcgctccaagctgggctgtgtgcacgaaccccccgttcagcccgaccgctgcgc
cttatccggtaactatcgtcttgagtccaacccggtaagacacgacttatcgccactggcagcagccactggtaacaggattagcagagcgaggtatgtaggcggtgctacagagttcttgaa
gtggtggcctaactacggctacactagaagaacagtatttggtatctgcgctctgctgaagccagttaccttcggaaaaagagttggtagctcttgatccggcaaacaaaccaccgctggtag
cggtggtttttttgtttgcaagcagcagattacgcgcagaaaaaaaggatctcaagaagatcctttgatcttttctacggggtctgacgctcagtggaacgaaaactcacgttaagggattttggt
catgagattatcaaaaaggatcttcacctagatccttttaaattaaaaatgaagttttaaatcaatctaaagtatatatgagtaaacttggtctgacagttaccaatgcttaatcagtgaggcacctatc
tcagcgatctgtctatttcgttcatccatagttgcctgactccccgtcgtgtagataactacgatacgggagggcttaccatctggccccagtgctgcaatgataccgcgagacccacgctcacc
ggctccagatttatcagcaataaaccagccagccggaagggccgagcgcagaagtggtcctgcaactttatccgcctccatccagtctattaattgttgccgggaagctagagtaagtagttc
gccagttaatagtttgcgcaacgttgttgccattgctacaggcatcgtggtgtcacgctcgtcgtttggtatggcttcattcagctccggttcccaacgatcaaggcgagttacatgatcccccat
gttgtgcaaaaaagcggttagctccttcggtcctccgatcgttgtcagaagtaagttggccgcagtgttatcactcatggttatggcagcactgcataattctcttactgtcatgccatccgtaaga

tgcttttctgtgactggtgagtactcaaccaagtcattctgagaatagtgtatgcggcgaccgagttgctcttgcccggcgtcaatacgggataataccgcgccacatagcagaactttaaaagt
gctcatcattggaaaacgttcttcggggcgaaaactctcaaggatcttaccgctgttgagatccagttcgatgtaacccactcgtgcacccaactgatcttcagcatcttttactttcaccagcgttt
ctgggtgagcaaaaacaggaaggcaaaatgccgcaaaaaagggaataagggcgacacggaaatgttgaatactcatactcttcctttttcaatattattgaagcatttatcagggttattgtctc
atgagcggatacatatttgaatgtatttagaaaaataaacaaataggggttccgcgcacatttccccgaaaagtgccacctgacgtctaagaaaccattattatcatgacattaacctataaaaat
aggcgtatcacgaggccctttcgtctcgcgcgtttcggtgatgacggtgaaaacctctgacacatgcagctcccggagacggtcacagcttgtctgtaagcggatgccgggagcagacaag
cccgtcagggcgcgtcagcgggtgttggcgggtgtcggggctggcttaactatgcggcatcagagcagattgtactgagagtgcaccatatgcggtgtgaaataccgcacagatgcgtaa
ggagaaaataccgcatcaggcgccattcgccattcaggctgcgcaactgttgggaagggcgatcggtgcgggcctcttcgctattacgccagctggcgaaagggggatgtgctgcaaggc
gattaagttgggtaacgccagggttttcccagtcacgacgttgtaaaacgacggccagtgaattcgagctcggtacCGGAGGATGGCAGCGTGATTTCACGGTCG
AGCGTCAGCGTCCGGGTCTGGCTGTTCACCGCCAGCACACGACCACCGGTGCTGATACCGGCATAGTCATCATCGC
AGATTTCAATAACATCGCCCGGTACATGGCGAAGCCCTTCTGCGCCGACGCTGAAATCCACGGTCTGCGTTTCCAG
CAGTTCTGTTTTAATCAGCCACAGCCCGGCGCGGTGTGCCTGCCCCCGGCTGGTACAGCCAAAGGCATCCATCTTC
GTAACATTACGACCGTAACGGGCAATGGCCTGCGTATCTTCAACAAGCTCTGTCGCCGTCTCCCAGCCGTTGTTCG
GGTCAATCCAGTTCACCTCAACGGCATTATGGCGGTCCTTCAGGGCGCTGAAGCTGTAGCGGAACGGCGCGCCATC
ATCCGGCATCACCACATTACTGCGGTTATAGGTCCACGTCTTATCCGACGGTCGGTCCTGCACGAACGTCAGCGTC
TGCCCGTTCCATACCGGCATACAGCGCATCGCCGAGCAGAAATCGCTGAGCACATCCCACGCCTTACGCTGTGTGG
TCAGGTACGCATTACAGGTGATGCGCGGCTCCGTGCCGCCAAAGCCGTCCGGCACTGACTGGTCGCAGTACTGGCC
GATGACATACAGCGCCCATTTATCCACATCCGCCGCACCAAGACGTTTCCCCATGCCGTAGCGCGGATGGGTCAGC
ATATCCCACAGACACCAGGCCATGTTGTTGCTGTATGCCGGTTTAAACGTTCCGTCCCAGATACCGCTGTATTGCCG
CGTCTGCGGGTTATAGTTCGACGGCACCTGCAGAATACGCCCGCGCAGATGATAATTACGGCTCACCTGCTGGCTG
CCGAACTGCTCCGAGTCCACCTGCACGCCGACCAGTGCCGTGTTCGGGTAGCACTGTTTCACATCGATGATTTCAG
TGTATGACGACCAGAGCGTTTTGTTCTGCAGCTGGTCTGTGGTGCTGTCCGGCGTCATCCTGCGCATCCGGATATTA
AACGGGCGCGGCGGCAGGTTACCCATCACCACCGAGGCCAGATACTGCGAGGTGGTTTTGCCCTTAATGGTGATGT
CTTTTTCCGTCACCCAGCCACCGTTACGTTGTATCTGAACCAGCAGGCGGACTTCCGACGGATTCCTGTCACCCTTT
GAGGTGGTTTCCACCAGTGCCTGTACACCGAAGGTAAAGCGCAGACGGTCGATGTTTGCAGACGTAATGGTGCGG
GTGATCGGCGTGTCATATTTCACTTCCGTACCCAGCACCGTCTCGGAGCCGGAGGATTCAAATCCCTCCGGCGGAG
TCTGCTCCTGCTCACCAGCCCGGAACACCACCGTGACACCGGATATGTTGGTATTCCCCTCAGTGTCCAGCACCGG
CGTACTGTTCAGCAGCACGCTTTTTAAGCCATCCACCGGACCTTCAATCGGCCCTTCGCTGATGGCATCGATCACA
CTCAGCAACTGCGTGGACTTCAGGTTGTCCTTCGCTTCGCGCGGGGTATGCCCCTTACTGCTTCCTTTACCCATTCC
TCACGCTCCATAAATGACAAAACCGCCCGCAGGCGGTTTCACATAAAACATTTTGCATCAGCGACCAATCACCACA
ACCTGACCACCGTCCCCTTCGTCTGCCGTGCTGATCTCCTGAGAAACCACGCGTGACCCCACGCGCATTTCCCCGT
ACAGAACAGGCAGAACATTGCCCTGGGCAACCATGTTATCCAGTGAGGAGAAATAGGTGTTCTGCTTACCGTTATC
CGTTGTCTGTATACGGGGAGTTCTGGCTTTCGGTGCCAGCATCTGCGCCACACCACCGAGCACCATACTGGCACCG
AGAGAAAACAGGATGCCGGTCATACCACCGGCCCCAATGGCTGCCCCCCATGCTGCAAGGGTGGCTCCGGCGGTA
AAGAATGATCCGGCAATGGCGGCAGCCCCCAGGACAATCTGGAATACGCCACCTGACTTGGCCCCGGCGACTCTG
GGAACAATATGAATTACAGCGCCATCAGGCAGAGTCTCATGTAACTGCGCCGTTAACCCGGACGTGCTGACGTCC
CGCCCGGCAATCCGTACCTGATACCAGCCGTCGCTCAGTTTCTGACGAAACGCCGGGAGCTGTGTGGCCAGTGCCC
GGATGGCTTCAGCCCCCGTTTTCACACGAAGGTCGATGCGGCGACCAAATCGTTGTAAATCCCCGTAAAGGCAGAT
GCGCGCCATGCCCGGTGACGCCAGAGGGAGTGTGTGCGTCGCTGCCATTTGTCGGTGTACCTCTCTCGTTTGCTCA
GTTGTTCAGGAATATGGTGCAGCAGCTCGCCGTCGCCGCAGTAAATTGCGGCGTGATTCGGCACTGATGAACCAAA
ACAGCACAGCAGCACATCGCCCGGCTGTGCCGCTGACAACGGCACCTGATACAGCCCCGTCGCCTCCAGATTATCC
AGATAGAGATTCTGGCCGTTACGCCACCAGTCATCCTCACGATGAAAGTCCGGCATCTCAATCCCCGCCAGATGAT
AAGCATCCCGGAACAGTGTGTAACAGTCCGTCACACCGTGCTCAAAGCGCCGCCCGGTGAGATGCGGCACACAGC
GGAACTTATGAATCGTCCCCCGGCAGACCAGCCACCACGGCAAATCACTCTGCACCTGCAGCCGCCGGTCGGCCTC
ACTCAGCCAGGGCAGACCACCGGGGTGGCTGTGGACCAGCGCCACAATCTCACCCTGCATTTCTGCCTGCAGCCA
GTCTTCCGGCGACATACGGAAATAGCCTCCGGCTCACCGGAGATATTCACGCAGGGGAAATATCTTTCCCCCTCCG
GCGTGCTTACCACGAAGCCGCACGACTCCGCTGGCGCACATCGCCGGGCGTGCGCCAGAATCGCTGATTCTGTCTG
TGTCATGGGATTTACTGCGAAAGTTTGTTAATGGAAAGGAAGCCGCCAAAGTTGCCGACGTTATTGCGGAACTTAC
AACCGCTCAGGCATTTGCTGCATTTATCCTTCGTGATATCGGACGTTGGCTGGTCATATTCATCCGCGACAGCCGGA
CCGCTATAACCGCACTCGTCACCGCGATAGGTCCAGGTGCAGGTGTTGGCCAGCATGATACGTCCCGGAAAAACA
GCGCCATCCGTTTCCGTCGGCGTGGACAGTACAAAGGAGGCACTCACCGCGCTCAGTTCGCTGCACTGCTCAATGC
GCCAGCGGCTGATCACCTCCTGCTCCGGATCGGCGTAACTGTTTCCGTTGACGAAGTTCACCGCATCCAGAAAACG
GGCGTAAACCTTACGCCGGACCACCGTTCCGCCGACCAGACTCTGCATATCTTCCGCCATCCCGGTGACCATACCG
TACAGGTTAGAAACCGTCAGCGTGGGGCGCGTACTGGTGCCTTTGCCATTCAGTTCAAAACCGCTCCCCTGAATGG
GATACGGCTGATACTGTCGCCCCTGCCAGGTGACCGGCTCACCTTTTTCGTTCTGCTCATTACAGAAAAAATAACG
TTCTCCACCGACCTCTGTCAGGTCGATTTCCCAGAGCACCACGCTGGCCGACTGCTCCGCACGGGTGCATTCATTC
AGTGTTTCCTGCCGGATATCCTGCATCAGTTCACCACCTGTTCAAACTCTGCGCTGAACTCAACACGCAGCATACT
GACCCGCGACGACCATTTTGCGCAGGTCACCTTTATCTGCCGCCACTCATAAGGCGGCGTCCACAGAAAGGATTTC
CAGCCCCCGTGCTCTTCCAGAAACGACTCCAGTACCGTGGCCTCCTCACGGGGGACAGAAAGCGTCACGCTGTAC
GTTTTCAGGTTGGCATTCAGCCCGGCAGGCGCTCGCTGAGAATAGCCATCACCAAAGCGCACCTTTCTTACAGAAG
GGACCGAAGCCACATCCATACCGGGTTTCACTTTCCAGCGGAAGGTCTTCATCGTCCACCTCCGGAGAACAGGCCA
CCATCACGCATCTGTGTCTGAATTTCATCACGGGCACCCTTGCGGGCCATGTCATACACCGCCTTCAGAGCAGCCG
GACCTATCTGCCCGTTCGTGCCGTCGTTGTTAATCACCACATGGTTATTCTGCTCAAACGTCCCGGACGCCTGCGAC

CGGCTGTCTGCCATGCTGCCCGGTGTACCGACATAACCGCCGGTGGCATAGCCGCGCATCAGCCGGTAAAGATTCC
CCACGCCAATCCGGCTGGTTGCCTCCTTCGTGAAGACAAACTCACCACGGTGAACAATCCCCGCTGGCTCATATTT
GCCGCCGGTTCCCGTAAATCCTCCGGTTGCAAAATGGAATTTCGCCGCAGCGGCCTGAATGGCTGTACCGCCTGAC
GCGGATGCGCCGCCACCAACAGCCCCGCCAATGGCGCTGCCGATACTCCCGACAATCCCCACCATTGCCTGCTTAA
GCAGAATTTCTGTCATCATGGACAGCACGGAACGGGTGAAGCTGCGCCAGTTCTGCTCACTGCCGGTCAGCATCGC
CGCCATATTCTGTGCAATACCATCAAAGGTCTGCGTGGCTGCACTTTTTACCTGCGACATACTGTCCGTGGCGCTCT
CTTCCCACTCACTCCAGCCGGACTTCAGGCCTGCCATCCAGTTCCCGCGAAGCTGGTCTTCAGCCGCCCAGGTCTTT
TTCTGCTCTGACATGACGTTATTCAGCGCCAGCGGATTATCGCCATACTGTTCCTTCAGGCGCTGTTCCGTGGCTTC
CCGTTCTGCCTGCCGGTCAGTCAGCCCCCGGCTTTTCGCATCAATGGCGGCCCGTTTTGCCCGTTGCTGCTGTGCGA
ATTTATCCGCCTGCTGCGCCAGCGCGTTCAGGCGCTCCTGATACGTAACCTTGTCGCCAAGTGCAGCCAGCTGGCG
TTTGTACTCCAGCGTCTCATCTTTATGCGCCAGCAGGGATTTCTCCTGTGCAGACAGCTGGCGACGTTGCGCCGCCT
CCTCCAGTACCGCGAACTGACTCTCCGCCTTCCACAAATCCCGGCGCTGCTGGCTGATTTTCTCATTTGCTCCGGCA
TGCTTCTCCAGCGTCCGGAGTTCTGCCTGAAGCGTCAGCAGGGCAGCATGAGCACTGTCTTCCTGACGATCGCCCG
CAGACACCTTCACGCTGGACTGTTTCGGCTTTTTCAGCGTCGCTTCATAATCCTTTTTCGCCGCCGCCATCAGCGTG
TTGTAATCCGCCTGCAGGATTTTCCCGTCTTTCAGTGCCTTGTTCAGTTCTTCCTGACGGGCGGTATATTTCTCCAGC
GGCGTCTGCAGCCGTTCGTAAGCCTTCTGCGCCTCTTCGGTATATTTCAGCCGTGACGCTTCGGTATCGCTCTGCTG
CTGCGCATTTTTGTCCTGTTGAGTCTGCTGCTCAGCCTTCTTTCGGGCGGCTTCAAGCGCAAGACGGGCCTTTTCAC
GATCATCCCAGTAACGCGCCCGCGCTTCATCGTTAACAAAATAATCATCCTTGCGCAGATTCCAGATGTCGTCTGC
TTTCTTATACGCAGCCTCTGCCTTAATCAGCATCTCCTGCGCGGTATCAGGACGACCAATATCCAGCACCGCATCCC
ACATGGATTTGAATGCCCGCGCAGTCCTGTCTGCCCAGGTCTCCAGCGTGCCCATGTTCTCTTTCAGGCGGCGGGT
CTGGTCATCAAACCCTTTCGTTGCGGCCTCGTTCGCCGCCTGCAATGCCCCGGCTTCATCGCCGGAACGCTGCAACT
GAGCAACATACGCAATCTGCTCCGCCGACACGTTATGGAACTGGCGAGCCATCGCCGTCAGCCCCGACGTCGGGT
CTGTGGTCAGCTTCCCGAAGGCTTCAGCGACCTTGTCCACCTCCACGCCGGATGCAGAGGAGAAACGCGCCACACT
CTGGCTGATGGACGCAATCTGAGCCTCACCGCTTACCCCCGCCTTAACCAGTGCGCTGAGTGACTCGCTGGTCTGG
TTAAACGTCAGCCCTGCCGCCTGCCCGGCTCTGGACAGGACCAGCATACGATCTGCCGTCAGTCCCGCCTGATTGC
CGGAAAGGACCAGCGTTTTGTTGAAATCGGACAGGGTTGAGTTGCCCTGATACCAGGCATACGCCAGCGCACCGG
TCGCCACCGCCAGCGAGGTGGCCCCCACCATCGGCAGGGTGATCGCACCGGCAAGCCCCCTGAACATGGGGATCA
TCCCGCCGAAGGAGTCCTTCACCTGCCCCCCCTGTTGCAGCAGGATCAGCCACGGACTTTGCCCGCCTGCAAGCTG
CGTGGCCACGTCGGTGAACTGTGCAGGCAGCATACGCATGGCGGCTTTATACTGCCCGACGGAAATCCCCGCTTTC
TGTGCAGCCAGCGCCTGTCGGCTCAGCGACTGTTCAACGACTGCCGCTGTTTTTTTCGCATCACTTTCCGTACCAGA
AAAATGACGCCTGACTCTGGCCATCTGCTCGTCAAATCTGGCCGCATCCAGACTCAAATCAACGACCAGATCGCCT
ACCGGTTCAGCCATACCGGACTCCTCCTGCGATCCCTTCTGATACTGTCATCAGCATTACGTCATCCTCCGTCATGT
CCGCCACATCCGGGGAAGCGGGGATAACTTCATTCCCGTCCGGGCCAAAGCGGACACCTCCGGCAAGCCCTGCCG
CTTTCTGCATCAGCACATCATCTTCAGGCTCTTCGTCAGCCTCGCGCCGGTTCAGCAGACTGAAATCCAGCGGATG
CATATCCGGATCGCTGAAAAACAGGCTGAGCACGGTGTACGTCAGCCCGGAAAAGTGCATATCCAGCAGAACATC
ATGAAAATAATGGGTACTGTAAAAGCGGTGCCAGTCGGCATACTCCGTGGATGACATCCCGGCAAGCATGGCACG
CCAGTCGGGTCGCCCCATCTCACGCGCCAGTTTCAGGGCAAAACTCAGCTCACCGTCGAACACTTTCCCGCAGAAA
CAGGCTCTGCGGGCCCGGCGTCCTCTGTCTGTTCAGGGGCATTATTCACCACAAACTCATACATACCAGACAGCCG
GTACACCACGTTTTCAGCATGAGAAATTGCCTCCGTGGGCCAGGTGGTAAGCACTTCCTGCTCAATCTGTTTAACG
GCTTCATTCATGGACGGCATCTGCGTCTTCTGCGGATGGTTATGCCACAGGGACATCGCCACCAGAAACGCGCCGG
TTCTGATGGCGTCTTCCACAGTAAACTTCCGGTTGCTGTCTGACTCCGCCTGTTCTGCCTGCCGTTTCATCAGGGCG
AGATGCTCAATGCGCTGCAGGGCTGACAGTTCAGAAAGCGTGACGGTCACACCGTTATGTTCAAATGATTCGGTTT
TCAGGAACATCGCTGACTCTCCGGATTAACTGGCGGTGACGGTAATTTCTGCAACCGCAGCAAACTCACCATTACC
GGATACAACCGGAATGTTGACCTTGCCTGCAGCAACGCCGTTCACGGTGATGGTCATACCACTGACCGACACGGT
GGCTTTTGTTTTATCCGCAGACACCGCACGAAAGCTCTTGTCGGTTACGCCCTCCGGCTGGAAGGCCACGGTCAGC
GTGGTGCTCTGCCCTTTCACCACCGAGGTGCTGGCAGGCGTCACGGTCATGCCGGTTGCCGCTGTTACCGTGCTGC
GATCTTCTGCCATCGACGGACGTCCCACATTGGTGACTTTCACCGTGCGGGTGATCACTTCCTTCGCCGTCACCGCC
TTACCGATACTGCTGACCCAGCCACGGAACACATCGACCGTGCCGTTCGGGAAGCGGATTTTATAGGCACGGGTAT
CGCCTTCATTAAACCACGCCAGCAGCGCCTGCTGCCCCTGCTCTCCGGGCATCCACGCCAGCGTGAAGCTGGTATC
TCCGGCAGATTTCTGCCCCTGCCCGGTCGCAGTCCAGTCTGCATCTTCATCATCGAGATAGCTGTCGTCATAGGACT
CAGCGGTCAGTTCGCCGGGCGTCAGGTCTTTAACTTTTGCCAGACGCGACCAGTCAACGTCTGAAAGCGGATTCGC
GTAAGGGTCACCGCTCCCCTTATAAACCCACAGGGTGGTCCCGGCACCTTTCACCGGCATTGTAGGATTTGGTACA
GGCATAGCGTCCTCACATTTCATAGGTAATGACATAAGTCAGATCGGCTGAACTCCACAAGCCCGCATCATCGTCG
CGCCGGTAGTCATAGCCGCTGGCCACCATACTGGTGATCAAATCTGACAGTGCCGGGATATCGCTCATCACCGGAT
AAATCCGGGACTCCATCCACGCATCCAGCTCTGAATCCGGCACCTGAGCAGGCAGGAAAACTTCGATATGCAGCT
CCGCCTGCCAGGTATCGCTGTCCAGCTCTTCGCCCGTGTATTCAGCGCCGGTGAGATAAACGGCAACTGCCGGAAA
ATCCGCCTCATCAAAAACAGCGGGGCGACCATCAAAAAACGTCGCCCCGGTGTCATGCTTCTCCAGTGCATCCAGT
ACGGCTGCACGGAGTTCAGTATGTTTCATCGCTTTATTACCATCCTCAGTTGATGCTGCAGCGCATAGCCCAGCTCT
TTCGGAAGACGTTCACGCCGTATCCGCTCAATATTTTGTTTAAACGCCGTGGTCAGCGGCACCGCCATCGGGATTTT
CACCACATCAATGGGGTAACGGTTTTTCCCAGCCACACGCTGCATGACATGCCACCGGCCATTTTTCAGTTGCTGA
ATAAACGCGCCGGGAATACGACGGTTACCCACCACAAGCACGCTGCCGCCACCTTTCAGGGATGAACGCTGCCCC
TTTTTACGACGCCTGCGGCGCGAAAGGACAACCCGCGCATTACCCAGCTTGATTACGGGCAAATCCCCCCGGTTAA

CTTTGATTCTGGCCTGCGGATTTTTGACCGTGGCCCTTTTCAGCCTGGCCCTTTCCTTTACCAGTTTCCGGCGTACCT
TTGTCTCACGGGCAACCTGTGACGCCGACTGCGATATCGCGGATGAAGCAACGCGGTTAATGGCCATTGCGGCGG
CACCAGGCACCGCCGTTTTGCTGATACGGCTGAGGTTTTCAACGGCCTGCTCAAGACCTTTTATGGCCATACATCC
CCCTTTCAGCGGCGACGGTTAACGGCAGGCGGTACGCCCCGTCCAAGCCAGAGATGACAACTTCCGCCATCATCC
GGCGAAACCCGATCTACCCAGAAATTTTCCTCACCGATGGTCAGCGTGTCTCCACGCCGCAGCTGCCGCACCTCAT
CAGTCCGGACAAACAGGGACGGGCTGGAGCCTTCAACGCGCACGCCCTGTCCGGCATAGCTGATATTTTCAGGGT
CATCAAAAACACCACGTATCACCGCACCTGACTGCTCACCGGATGTAATGGTGGCTGACGTTCCCATGTACCCGCG
TATCGTTTCATCGGCGCGGGCAATGGCAGCATCGAACAGGTTATCGAAATCAGCCACAGCGCCTCCCGTTATTGCA
TTCTGGCCAGGCCGCGCTCTGTCATTTCGGCTGCCACACCGGCAGAGACACGAAACGCCGTTCCCGGCAGCACAA
ATGCCACAGGTTCATCCCGCGTGGCGTGAAGTGCATCAGTATGCAGCTTCACCAGTGCCACGACCGTGACCAGTTC
AGACGTATCCAGAATCACGGTATCCGGCTGCGCTGATCCCACCTCATTTTCATGTCCGGTCAGCACATTTTCCCGGC
TGAGAGGGGTGTCCTGACCGGCAGTTTCATCCGTGTCATCAAGCTCCTCTTTCAGCTCTGCCACACGGAGCGCCAG
TTCTTCTTTCGTCCCCGTCAGGCTGACATCACGGTTCAGTTGTTCACCCAGCGAGCGGAGACGGGCAATCAGTTCAT
CTTTCGTCATGGACTCCTCCACAGAGAAACAATGGCCCCGAAGGGCCATGATTACGCCAGTTGTACGGACACGAA
CTCATCAGGGTCAGCCAGCAGCATCAGCGGTGCTGACTGAATCATGGTGAACTCACGCGCCGGATCGCCGGTGGT
CACCCAGTTTTTCGGGTAACGGGCAGAGGCGTTAATGCCTTCGCGCTGTGCGTCCGCATCCTGAATGCAGCCATAG
GTGCGCAGACCGCGTGCCTGAGTGTTCCCCAGCACCATCGTGTTGTCCGGCAGGAAGTTCTTTTTGACGCCGTTTTC
CACGTACTGTCCGGAATACACGACGATGGCCACATCGCCATACATCCCCTTATAGGACACCGCTTTGCCCAGGTCT
TTCACCGCTGTCTCCAGCTCGGAATTAGAGCCACGACGGGTATCCAGCTTCTCCTTGACGGCTTTGAAGGAACGGA
ACAGCGCCCAGCCTTTCGGATCGAACACGATGATATTCACCACACCGCTGGCGTTCAGCGCGTAGGCTTCGATATC
GTCGGTCGGGTCATACGTGGACTTGTCACGCTTGCTCCACTCCGTGCCGCCGGACTGCGTGATGTTATTCTCCTCAC
TGCGGCCCATATCCACCTCAACCGGATCGAAGGCTTCACCGGTCATGGTGTATTTGCCCTTAAGCACGGCAGAAAC
TGCCTGCATCTCTTCGACCTGAGCAATGGCCAGCTCTTCGTCACGCATGTTCTGCATGATGATGCGACGGCGGCGG
TAAGCCGGGTCCGCCAGATTCTGCGGATCTTCATCCGGCAGGCGACGCAGGGTCATCTGCGGATTCACTTCATGCT
TCGGCTTGACATATCCCGGCGTAAATTCAGAGGTGGAGCCGCCACGGGAACGGATAACCTCACCGGAAACAATCG
GCGAAACGTACAGCGCCATGTTTACCAGTCCCGGAATTTGTGAGAGATAGACTTTCTCCGTGGTGAAGGATAGCT
CTCACGGAAAAAGAGACGCAGAAACAGCGGATCAAACTTAAATTTCTGCTCATTTGCCGCCAGCAGTTGGGCGGT
TGTGTACATCGACATAAAAAAATCCCGTAAAAAAAGCCGCACAGGCGGCCTTTAGTGATGAAGGGTAAAGTTAAA
CGATGCTGATTGCCGTTCCGGCAAACGCGGTCCGTTTTTTCGTCTCGTCGCTGGCAGCCTCCGGCCAGAGCACATC
CTCATAACGGAACGTGCCGGACTTGTAGAACGTCAGCGTGGTGCTGGTCTGGTCAGCAGCAACCGCAAGAATGCC
AACGGCAGCACCGTCGGTGGTGCCATCCCACGCAACCAGCTTACGGCTGGAGGTGTCCAGCATCAGCGGGGTCAT
TGCAGGCGCTTTCGCACTCAATCCGCCGGGCGCGGTTGCGGTATGAGCCGGGTCACTGTTGCCCTGCGGCTGGTAA
TGGGTAAAGGTTTCTTTGCTCGTCATAAACATCCCTTACACTGGTGTGTTCAGCAAATCGTTAACGGCATCAGATGC
CGGGTTACCTGCAGCCAGCGGTGCCGGTGCCCCCTGCATCAGACGATCCAGCGCAGTGTCACTGCGCGCCTGTGCA
CTCTGTGGTGCTGCGGCCAGAATGCGGCGGGCCGTTTTCACGGTCATACCGGGGGTTTCTGCCAGCACGCGTGCCT
GTTCTTCGCGTCCGTGAGCCTCCTCACAGTTGAG

Plasmid (pMDW111) Sequence

tcgcgcgtttcggtgatgacggtgaaaacctctgacacatgcagctcccggagacggtcacagcttgtctgtaagcggatgccgggagcagacaagcccgtcagggcgcgtcagcgggt
gttggcgggtgtcggggctggcttaactatgcggcatcagagcagattgtactgagagtgcaccatatgcggtgtgaaataccgcacagatgcgtaaggagaaaataccgcatcaggcgcc
attcgccattcaggctgcgcaactgttgggaagggcgatcggtgcgggcctcttcgctattacgccagctggcgaaagggggatgtgctgcaaggcgattaagttgggtaacgccagggttt
tcccagtcacgacgttgtaaaacgacggccagtgAATTCGCCTGGGTGGCTTCATTCGTTCTTTTGTTCCTTATTTTGTTCCTTACTTAGT
TGGTTATTTGCTTGTTTGGTTATTTATTTCGTTGGTTATTTGGTTAATTCCTTCTTTGCTTTCTTCATTCCTTTCTTGCT
TTATTCCTTGTTTTTTTGGTTTCTTAGTTTCCTTTTCCCTAGAGGTAGCCAAAGTCTTTGCAACTATACTTTCAGCTCT
GACAAATTTGTTCTTATTACTTCTCTTTTTTTTGATTTGTTCTTCCCTCTTTTTCTTAGCTAATTCTTGTCTTTCGATTC
TAGTTCTATCAGCATTTCTTTATAAATCTATTTTTTTTTTTTTTTTTCGACACAAAATGTCTATTTCTTGGAGTGCTTACT
CTTCTTTTTGTTTTTACCTTGTTTCAACTCGTTTAATCTATCAACTTTTTCCTTGATCCTTTCCAAAGATAATTTGACA
TCACCTTTTTGGCACTAGGTGCCACCGATGTGGAagcttggcgtaatcatggtcatagctgtttcctgtgtgaaattgttatccgctcacaattccacacaacata
cgagccggaagcataaagtgtaaagcctggggtgcctaatgagtgagctaactcacattaattgcgttgcgctcactgcccgctttccagtcgggaaacctgtcgtgccagctgcattaatga
atcggccaacgcgcggggagaggcggtttgcgtattgggcgctcttccgcttcctcgctcactgactcgctgcgctcggtcgttcggctgcggcgagcggtatcagctcactcaaaggcgg
taatacggttatccacagaatcaggggataacgcaggaaagaacatgtgagcaaaaggccagcaaaaggccaggaaccgtaaaaaggccgcgttgctggcgtttttccataggctccgcc
cccctgacgagcatcacaaaaatcgacgctcaagtcagaggtggcgaaacccgacaggactataaagataccaggcgtttccccctggaagctccctcgtgcgctctcctgttccgaccct
gccgcttaccggatacctgtccgcctttctcccttcgggaagcgtggcgctttctcatagctcacgctgtaggtatctcagttcggtgtaggtcgttcgctccaagctgggctgtgtgcacgaac
cccccgttcagcccgaccgctgcgccttatccggtaactatcgtcttgagtccaacccggtaagacacgacttatcgccactggcagcagccactggtaacaggattagcagagcgaggtat
gtaggcggtgctacagagttcttgaagtggtggcctaactacggctacactagaagaacagtatttggtatctgcgctctgctgaagccagttaccttcggaaaaagagttggtagctcttgatc
cggcaaacaaaccaccgctggtagcggtggtttttttgtttgcaagcagcagattacgcgcagaaaaaaaggatctcaagaagatcctttgatcttttctacggggtctgacgctcagtggaac
gaaaactcacgttaagggattttggtcatgagattatcaaaaaggatcttcacctagatccttttaaattaaaaatgaagttttaaatcaatctaaagtatatatgagtaaacttggtctgacagttacc
aatgcttaatcagtgaggcacctatctcagcgatctgtctatttcgttcatccatagttgcctgactccccgtcgtgtagataactacgatacgggagggcttaccatctggccccagtgctgcaat
gataccgcgagacccacgctcaccggctccagatttatcagcaataaaccagccagccggaagggccgagcgcagaagtggtcctgcaactttatccgcctccatccagtctattaattgtt
gccgggaagctagagtaagtagttcgccagttaatagtttgcgcaacgttgttgccattgctacaggcatcgtggtgtcacgctcgtcgtttggtatggcttcattcagctccggttcccaacgat
caaggcgagttacatgatcccccatgttgtgcaaaaaagcggttagctccttcggtcctccgatcgttgtcagaagtaagttggccgcagtgttatcactcatggttatggcagcactgcataatt

ctcttactgtcatgccatccgtaagatgcttttctgtgactggtgagtactcaaccaagtcattctgagaatagtgtatgcggcgaccgagttgctcttgcccggcgtcaatacgggataataccg
cgccacatagcagaactttaaaagtgctcatcattggaaaacgttcttcggggcgaaaactctcaaggatcttaccgctgttgagatccagttcgatgtaacccactcgtgcacccaactgatct
tcagcatcttttactttcaccagcgtttctgggtgagcaaaaacaggaaggcaaaatgccgcaaaaaagggaataagggcgacacggaaatgttgaatactcatactcttcctttttcaatattatt
gaagcatttatcagggttattgtctcatgagcggatacatatttgaatgtatttagaaaaataaacaaataggggttccgcgcacatttccccgaaaagtgccacctgacgtctaagaaaccattat
tatcatgacattaacctataaaaataggcgtatcacgaggccctttcgtc

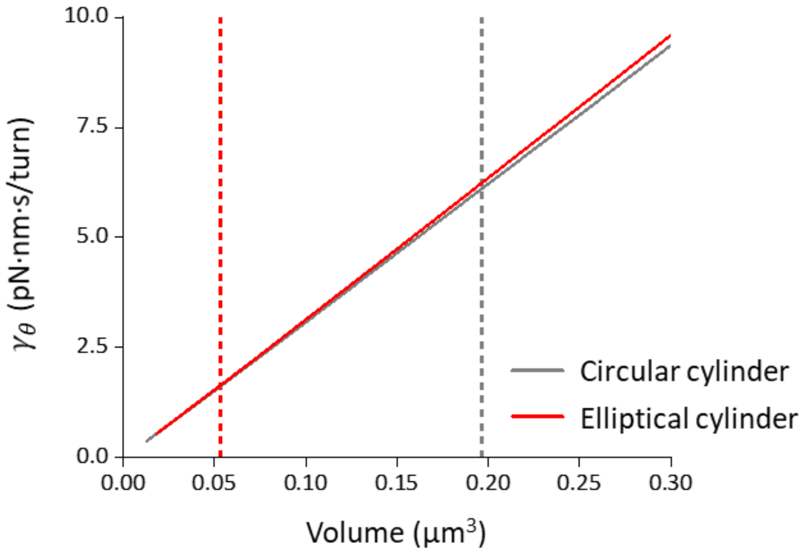

Fig. S1. Rotational viscous drag coefficients γ_θ of cylinders.
Simulated viscous drag coefficient γ_θ dependence on the cylinder volume with a fixed shape. For elliptical cylinders, the cross-section eccentricity ε is kept at 0.6, the cylinder aspect ratio $\frac{h}{2a}$ at 1.6. For circular cylinders, the cylinder aspect ratio $\frac{h}{D}$ is kept at 2. The red dashed line indicates the targeted volume of metamaterial elliptical cylinders to be fabricated. The grey dashed line indicates the typical size of quartz cylinders used for single-molecule studies(*19*, *22*).

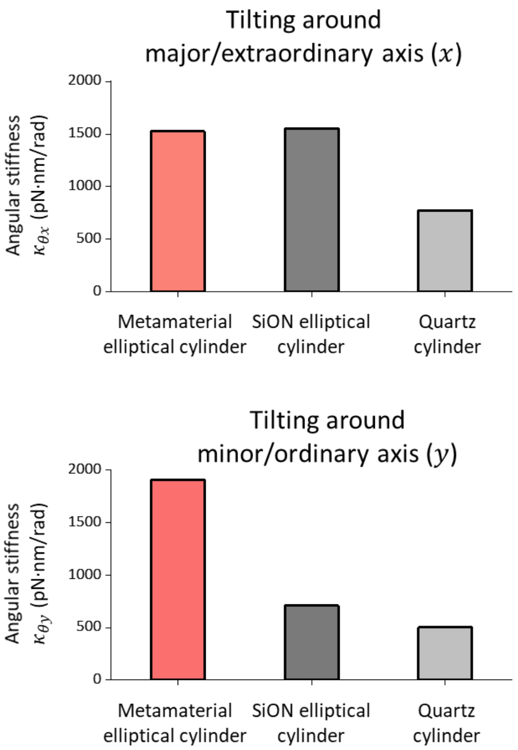

Fig. S2. Cylinder tilting in the trapping beam.

Simulated angular stiffness of metamaterial and SiON elliptical cylinders tilting around the major axis (x) and the minor axis (y). Also shown is simulated angular stiffness of a quartz cylinder tilting around its extraordinary axis (x) and ordinary axis (y). The volumes of these three types of cylinders are kept the same. The simulated angular stiffness characterizes how robustly cylinders align their cylindrical axis with the direction of the trapping beam propagation. The metamaterial (n_e = 1.66 and n_o = 1.75) and isotropic SiON (n = 1.75) elliptical cylinders both have the dimensions of ε = 0.6, $\frac{h}{2a}$ = 1.6, and h = 600 nm. The quartz cylinder (n_e = 1.54 and n_o = 1.53)(*66*) has the dimensions of $\frac{h}{D}$ = 1.8 and h = 600 nm.

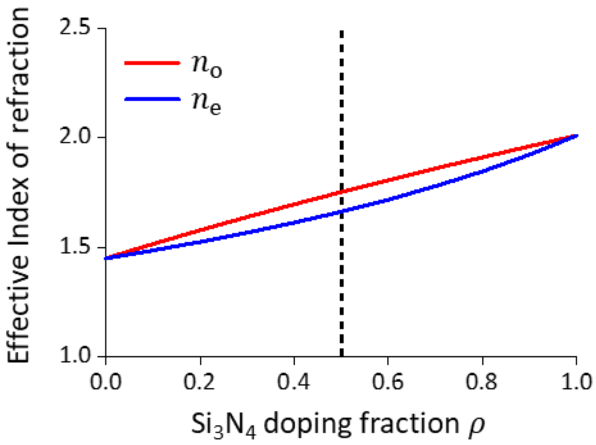

Fig. S3. The effective refractive index of the metamaterial doped with SiO_2 and Si_3N_4. The dashed line indicates the targeted doping fraction of the fabricated elliptical cylinders.

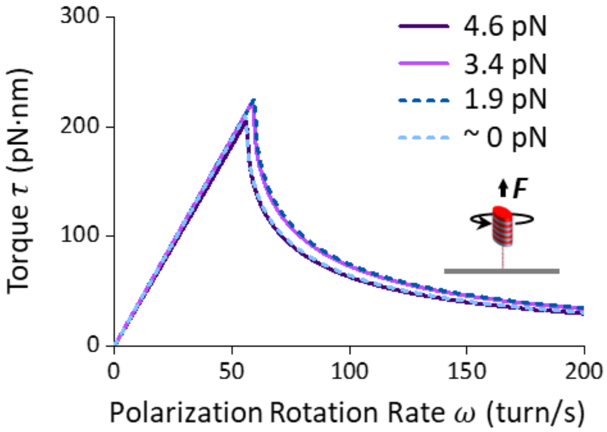

Fig. S4. Maximum rotation rate of metamaterial elliptical cylinders when the cylinder is displaced from the trap center.

Measurements were carried out in a similar way as in Fig. 4b, but with a metamaterial elliptical cylinder tethered to the surface via a torsionally unconstrained DNA molecule (inset)(*50*). As the cylinder was rotated, viscous drag torque was measured. Our measurements show that the metamaterial elliptical cylinder experiences an increase in maximum rotation rate at ~ 3 pN (corresponding to a distance of ~ 350 nm cylinder displacement from the trap center at 30 mW power before the objective). Notably, the slopes (viscous drag torque coefficient) of the linear regime of our measurements remain nearly constant, indicating our torque calibration remains unchanged when the cylinder is displaced from the trap center.

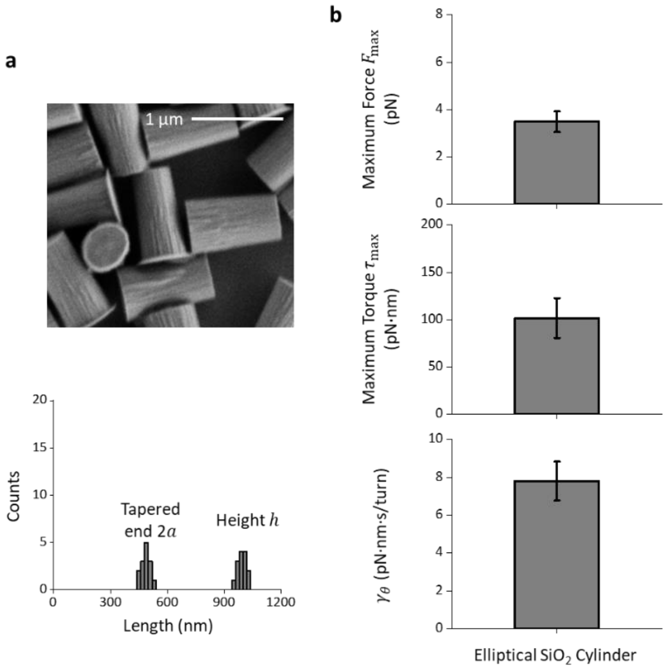

Fig. S5. Trapping properties of SiO_2 elliptical cylinders.

a. A scanning electron microscope image of nanofabricated SiO_2 elliptical cylinders. Scale bar: 1 µm. The cylinder dimension distributions are also shown.

b. Maximum trapping force F_{max}, maximum trapping torque τ_{max} at 30 mW laser power before the objective, and rotational viscous drag coefficient γ_θ of the SiO_2 elliptical cylinders (N = 14 cylinders). Values shown are mean $\pm$ s.d.

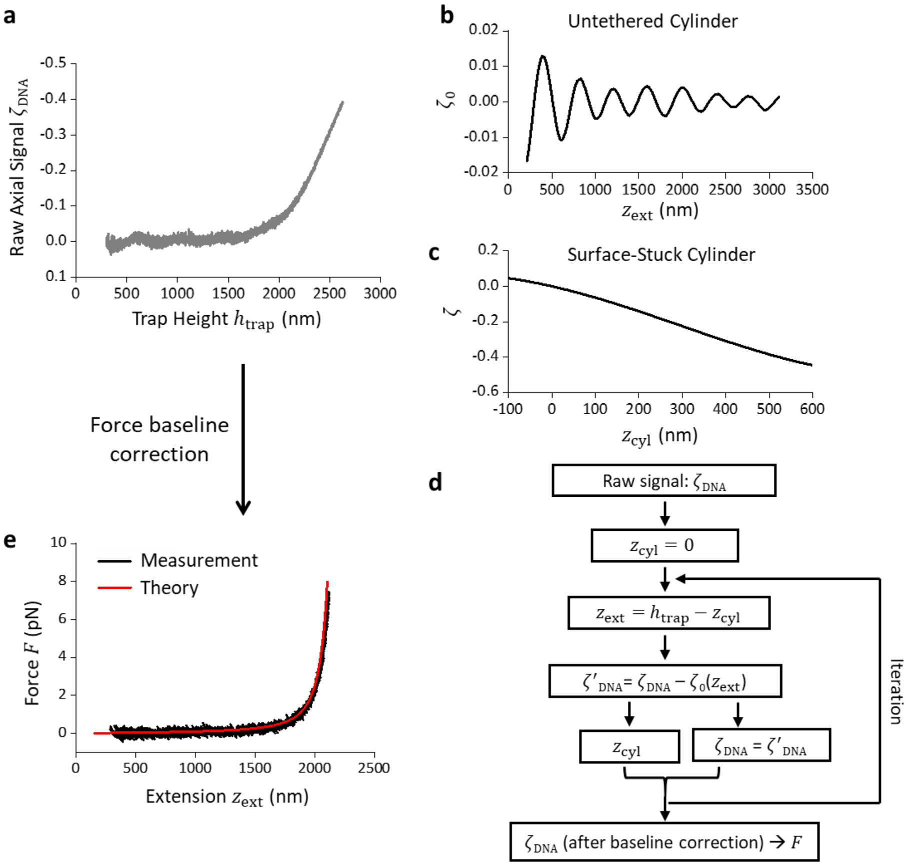

Fig. S6. An iterative algorithm for AOT force-offset correction for AOT measurements using the metamaterial elliptical cylinders.

The force to stretch a DNA molecule is measured via the normalized axial displacement detector signal ζ_{DNA} with an increase in the trap height h_{trap}: $F = k_z \cdot (-\zeta_{DNA})/S_z$, where k_z is the trap stiffness, and S_z is the axial displacement sensitivity(*21*). However, this raw signal contains a baseline due to the Fabry-Pérot effect that results from interference between the cylinder bottom surface and the sample chamber surface. The amplitude of the periodic force signal is negligible for the conventional quartz cylinder, whose refractive indices (n_e = 1.54 and n_o = 1.53) are comparable to the surrounding medium (n = 1.326). However, the high refractive indices of the metamaterial elliptical cylinder (n_e = 1.66 and n_o = 1.75) result in a much stronger interference and larger amplitude of the periodic force baseline (**a**).

To correct for this baseline, we performed the following procedure. We first measured the signal baseline ζ_0 of untether metamaterial elliptical cylinders to determine its dependence on the distance between the cylinder bottom surface and the sample chamber surface, which will corresponds to z_{ext} in a DNA stretching experiment (**b**). We next measured the signal ζ versus the cylinder displacement z_{cyl} by axially scanning surface-immobilized cylinders through the trap (**c**). Then, we used an iterative algorithm to convert the raw DNA-stretching data $\zeta_{DNA}(h_{trap})$ (**a**) to the corrected force versus extension data (**d**). The resulting force-extension curve agrees well with that prediction, validating this method (**e**). This method was implemented in the real-time DNA torsional measurements under a constant force (Fig. 5; Fig. S7).

a. Raw DNA-stretching data ζ_{DNA} versus h_{trap} using a metamaterial elliptical cylinder.

b. Signal baseline ζ_0 of untether metamaterial elliptical cylinders and its dependence on the distance between the cylinder bottom surface and the sample chamber surface, mimicking the end-to-end extension z_{ext} when being attached with a DNA molecule. Shown is an averaged baseline measured with $N = 6$ cylinders.
c. Signal ζ versus the cylinder displacement z_{cyl} by axially scanning surface-immobilized cylinders through the trap. Shown is an averaged relation measured with $N = 13$ cylinders.
d. Iterative algorithm implemented on the AOT for real-time signal correction to achieve precise force and extension measurements.
e. DNA force-extension curve after the force-offset correction, which agrees with the prediction using the modified Marko Siggia worm-like chain model(*67*, *68*).

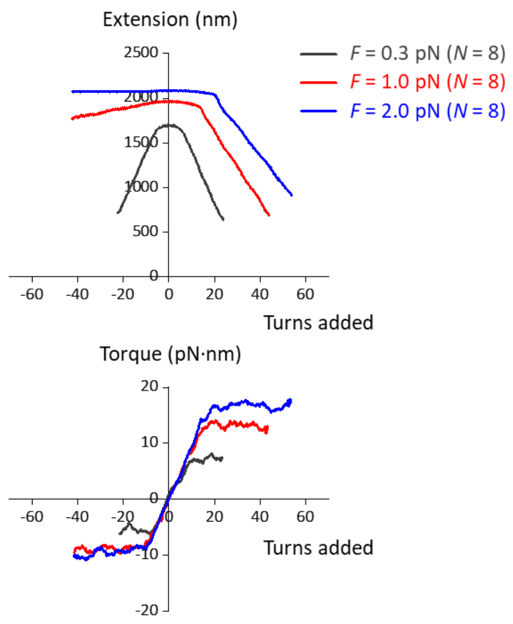

Fig. S7. DNA torsional measurements by metamaterial elliptical cylinders under different forces.

These measurements show that metamaterial elliptical cylinder can accurately measure the torsional properties of DNA over the standard force range for single-molecule experiments(*22*). The traces shown were averaged from individual measurements of N = 8 DNA molecules, where a sliding window of 0.1 turn was applied to average extension and a sliding window of 4 turns was applied to average torque.

REFERENCES AND NOTES

1. C. J. Bustamante, Y. R. Chemla, S. Liu, M. D. Wang, Optical tweezers in single-molecule biophysics. *Nat. Rev. Methods Primers* **1**, 25 (2021).

2. J. L. Killian, F. Ye, M. D. Wang, Optical tweezers: A force to be reckoned with. *Cell* **175**, 1445–1448 (2018).

3. P. M. Hall, J. T. Inman, R. M. Fulbright, T. T. Le, J. J. Brewer, G. Lambert, S. A. Darst, M. D. Wang, Polarity of the CRISPR roadblock to transcription. *Nat. Struct. Mol. Biol.* **29**, 1217–1227 (2022).

4. Z. Chen, R. Gabizon, A. I. Brown, A. Lee, A. Song, C. Diaz-Celis, C. D. Kaplan, E. F. Koslover, T. Yao, C. Bustamante, High-resolution and high-accuracy topographic and transcriptional maps of the nucleosome barrier. *eLife* **8**, e48281 (2019).

5. T. T. Le, Y. Yang, C. Tan, M. M. Suhanovsky, R. M. Fulbright, J. T. Inman, M. Li, J. Lee, S. Perelman, J. W. Roberts, A. M. Deaconescu, M. D. Wang, Mfd dynamically regulates transcription via a release and catch-up mechanism. *Cell* **172**, 344–357.e15 (2018).

6. B. Sun, M. Pandey, J. T. Inman, Y. Yang, M. Kashlev, S. S. Patel, M. D. Wang, T7 replisome directly overcomes DNA damage. *Nat. Commun.* **6**, 10260 (2015).

7. B. Sun, A. Singh, S. Sultana, J. T. Inman, S. S. Patel, M. D. Wang, Helicase promotes replication re-initiation from an RNA transcript. *Nat. Commun.* **9**, 2306 (2018).

8. L. Xu, M. T. J. Halma, G. J. L. Wuite, Mapping fast DNA polymerase exchange during replication. *Nat. Commun.* **15**, 5328 (2024).

9. M. J. Comstock, K. D. Whitley, H. F. Jia, J. Sokoloski, T. M. Lohman, T. Ha, Y. R. Chemla, Direct observation of structure-function relationship in a nucleic acid-processing enzyme. *Science* **348**, 352–354 (2015).

10. L. Guo, Y. Bao, Y. Zhao, Z. Ren, L. Bi, X. Zhang, C. Liu, X. M. Hou, M. D. Wang, B. Sun, Joint efforts of replicative helicase and SSB ensure inherent replicative tolerance of G-quadruplex. *Adv. Sci.* **11**, 2307696 (2024).

11. J. van Mameren, M. Modesti, R. Kanaar, C. Wyman, E. J. Peterman, G. J. Wuite, Counting RAD51 proteins disassembling from nucleoprotein filaments under tension. *Nature* **457**, 745–748 (2009).

12. J. J. Jiang, L. Bai, J. A. Surtees, Z. Gemici, M. D. Wang, E. Alani, Detection of high-affinity and sliding clamp modes for MSH2-MSH6 by single-molecule unzipping force analysis. *Mol. Cell* **20**, 771–781 (2005).

13. R. Anand, E. Buechelmaier, O. Belan, M. Newton, A. Vancevska, A. Kaczmarczyk, T. Takaki, D. S. Rueda, S. N. Powell, S. J. Boulton, HELQ is a dual-function DSB repair enzyme modulated by RPA and RAD51. *Nature* **601**, 268–273 (2022).

14. L. D. Brennan, R. A. Forties, S. S. Patel, M. D. Wang, DNA looping mediates nucleosome transfer. *Nat. Commun.* **7**, 13337 (2016).

15. M. Li, A. Hada, P. Sen, L. Olufemi, M. A. Hall, B. Y. Smith, S. Forth, J. N. McKnight, A. Patel, G. D. Bowman, B. Bartholomew, M. D. Wang, Dynamic regulation of transcription factors by nucleosome remodeling. *eLife* **4**, e06249 (2015).

16. Y. Cui, C. Bustamante, Pulling a single chromatin fiber reveals the forces that maintain its higher-order structure. *Proc. Natl. Acad. Sci. U.S.A.* **97**, 127–132 (2000).

17. L. F. Liu, J. C. Wang, Supercoiling of the DNA template during transcription. *Proc. Natl. Acad. Sci. U.S.A.* **84**, 7024–7027 (1987).

18. A. La Porta, M. D. Wang, Optical torque wrench: Angular trapping, rotation, and torque detection of quartz microparticles. *Phys. Rev. Lett.* **92**, 190801 (2004).

19. C. Deufel, S. Forth, C. R. Simmons, S. Dejgosha, M. D. Wang, Nanofabricated quartz cylinders for angular trapping: DNA supercoiling torque detection. *Nat. Methods* **4**, 223–225 (2007).

20. J. Ma, C. Tan, M. D. Wang, Single-molecule angular optical trapping for studying transcription under torsion. *Methods Mol. Biol.* **1805**, 301–332 (2018).

21. X. Gao, J. T. Inman, M. D. Wang, Angular optical trapping to directly measure DNA torsional mechanics. *Methods Mol. Biol.* **2478**, 37–73 (2022).

22. X. Gao, Y. Hong, F. Ye, J. T. Inman, M. D. Wang, Torsional stiffness of extended and plectonemic DNA. *Phys. Rev. Lett.* **127**, 028101 (2021).

23. J. Lee, M. Wu, J. T. Inman, G. Singh, S. H. Park, J. H. Lee, R. M. Fulbright, Y. Hong, J. Jeong, J. M. Berger, M. D. Wang, Chromatinization modulates topoisomerase II processivity. *Nat. Commun.* **14**, 6844 (2023).

24. S. Forth, C. Deufel, M. Y. Sheinin, B. Daniels, J. P. Sethna, M. D. Wang, Abrupt buckling transition observed during the plectoneme formation of individual DNA molecules. *Phys. Rev. Lett.* **100**, 148301 (2008).

25. M. Y. Sheinin, S. Forth, J. F. Marko, M. D. Wang, Underwound DNA under tension: Structure, elasticity, and sequence-dependent behaviors. *Phys. Rev. Lett.* **107**, 108102 (2011).

26. S. Forth, C. Deufel, S. S. Patel, M. D. Wang, Direct measurements of torque during holliday junction migration. *Biophys. J.* **101**, L5–L7 (2011).

27. M. Y. Sheinin, M. D. Wang, Twist-stretch coupling and phase transition during DNA supercoiling. *Phys. Chem. Chem. Phys.* **11**, 4800–4803 (2009).

28. J. Ma, L. Bai, M. D. Wang, Transcription under torsion. *Science* **340**, 1580–1583 (2013).

29. M. Y. Sheinin, M. Li, M. Soltani, K. Luger, M. D. Wang, Torque modulates nucleosome stability and facilitates H2A/H2B dimer loss. *Nat. Commun.* **4**, 2579 (2013).

30. J. Ma, C. Tan, X. Gao, R. M. Fulbright, J. W. Roberts, M. D. Wang, Transcription factor regulation of RNA polymerase's torque generation capacity. *Proc. Natl. Acad. Sci. U.S.A.* **116**, 2583–2588 (2019).

31. T. T. Le, X. Gao, S. H. Park, J. Lee, J. T. Inman, J. H. Lee, J. L. Killian, R. P. Badman, J. M. Berger, M. D. Wang, Synergistic coordination of chromatin torsional mechanics and topoisomerase activity. *Cell* **179**, 619–631.e15 (2019).

32. Z. Debyser, S. Tabor, C. C. Richardson, Coordination of leading and lagging strand DNA synthesis at the replication fork of bacteriophage T7. *Cell* **77**, 157–166 (1994).

33. D. C. Mace, B. M. Alberts, T4 DNA polymerase: Rates and processivity on single-stranded DNA templates. *J. Mol. Biol.* **177**, 295–311 (1984).

34. J.-B. Lee, R. K. Hite, S. M. Hamdan, X. Sunney Xie, C. C. Richardson, A. M. van Oijen, DNA primase acts as a molecular brake in DNA replication. *Nature* **439**, 621–624 (2006).

35. T. A. Baker, S. P. Bell, Polymerases and the replisome: Machines within machines. *Cell* **92**, 295–305 (1998).

36. T. M. Pham, K. W. Tan, Y. Sakumura, K. Okumura, H. Maki, M. T. Akiyama, A single-molecule approach to DNA replication in *Escherichia coli* cells demonstrated that DNA polymerase III is a major determinant of fork speed. *Mol. Microbiol.* **90**, 584–596 (2013).

37. M. M. Elshenawy, S. Jergic, Z.-Q. Xu, M. A. Sobhy, M. Takahashi, A. J. Oakley, N. E. Dixon, S. M. Hamdan, Replisome speed determines the efficiency of the Tus−*Ter* replication termination barrier. *Nature* **525**, 394–398 (2015).

38. D. Dovrat, D. Dahan, S. Sherman, I. Tsirkas, N. Elia, A. Aharoni, A live-cell imaging approach for measuring DNA replication rates. *Cell Rep.* **24**, 252–258 (2018).

39. J. T. P. Yeeles, A. Janska, A. Early, J. F. X. Diffley, How the eukaryotic replisome achieves rapid and efficient DNA replication. *Mol. Cell* **65**, 105–116 (2017).

40. C. Conti, B. Saccà, J. Herrick, C. Lalou, Y. Pommier, A. Bensimon, Replication fork velocities at adjacent replication origins are coordinately modified during DNA replication in human cells. *Mol. Biol. Cell* **18**, 3059–3067 (2007).

41. J. Inman, S. Forth, M. D. Wang, Passive torque wrench and angular position detection using a single-beam optical trap. *Opt. Lett.* **35**, 2949–2951 (2010).

42. K. C. Neuman, E. H. Chadd, G. F. Liou, K. Bergman, S. M. Block, Characterization of photodamage to *Escherichia coli* in Optical Traps. *Biophys. J.* **77**, 2856–2863 (1999).

43. U. Mirsaidov, W. Timp, K. Timp, M. Mir, P. Matsudaira, G. Timp, Optimal optical trap for bacterial viability. *Phys. Rev. E* **78**, 021910 (2008).

44. M. P. Landry, P. M. McCall, Z. Qi, Y. R. Chemla, Characterization of photoactivated singlet oxygen damage in single-molecule optical trap experiments. *Biophys. J.* **97**, 2128–2136 (2009).

45. S. Ha, Y. Tang, M. M. van Oene, R. Janissen, R. M. Dries, B. Solano, A. J. L. Adam, N. H. Dekker, Single-crystal rutile TiO_2 nanocylinders are highly effective transducers of optical force and torque. *ACS Photonics* **6**, 1255–1265 (2019).

46. Y. Tang, S. Ha, T. Begou, J. Lumeau, H. P. Urbach, N. H. Dekker, A. J. L. Adam, Versatile multilayer metamaterial nanoparticles with tailored optical constants for force and torque transduction. *ACS Nano* **14**, 14895–14906 (2020).

47. S. Ha, R. Janissen, Y. Y. Ussembayev, M. M. van Oene, B. Solano, N. H. Dekker, Tunable top-down fabrication and functional surface coating of single-crystal titanium dioxide nanostructures and nanoparticles. *Nanoscale* **8**, 10739–10748 (2016).

48. M. Peng, G. Xiao, X. Chen, T. Du, T. Kuang, X. Han, W. Xiong, G. Zhu, J. Yang, Z. Tan, K. Yang, H. Luo, Optical trapping-enhanced probes designed by a deep learning approach. *Photon. Res.* **12**, 959–968 (2024).

49. M. Riccardi, O. J. F. Martin, Electromagnetic forces and torques: From dielectrophoresis to optical tweezers. *Chem. Rev.* **123**, 1680–1711 (2023).

50. Y. Hong, F. Ye, J. Qian, X. Gao, J. T. Inman, M. D. Wang, Optical torque calculations and measurements for DNA torsional studies. *Biophys. J.* **123**, 3080–3089 (2024).

51. P. Ju, Y. Jin, K. Shen, Y. Duan, Z. Xu, X. Gao, X. Ni, T. Li, Near-field GHz rotation and sensing with an optically levitated nanodumbbell. *Nano Lett.* **23**, 10157–10163 (2023).

52. A. I. Bishop, T. A. Nieminen, N. R. Heckenberg, H. Rubinsztein-Dunlop, Optical application and measurement of torque on microparticles of isotropic nonabsorbing material. *Phys. Rev. A* **68**, 033802 (2003).

53. I. H. Malitson, Interspecimen comparison of the refractive index of fused silica. *J. Opt. Soc. Am.* **55**, 1205–1209 (1965).

54. K. Luke, Y. Okawachi, M. R. E. Lamont, A. L. Gaeta, M. Lipson, Broadband mid-infrared frequency comb generation in a Si_3N_4 microresonator. *Opt. Lett.* **40**, 4823–4826 (2015).

55. W. Cai, V. M. Shalaev, *Optical metamaterials : Fundamentals and Applications* (Springer, New York, 2010), pp. xii, 200 p.

56. Ľ. Podlucký, A. Vincze, S. Kováčová, J. Chlpík, J. Kováč, F. Uherek, Optimization of fabrication process for SiON/SiO_x films applicable as optical waveguides. *Coatings* **11**, 574 (2021).

57. C. Deufel, M. D. Wang, Detection of forces and displacements along the axial direction in an optical trap. *Biophys. J.* **90**, 657–667 (2006).

58. A. Jannasch, A. F. Demirörs, P. D. J. van Oostrum, A. van Blaaderen, E. Schäffer, Nanonewton optical force trap employing anti-reflection coated, high-refractive-index titania microspheres. *Nat. Photonics* **6**, 469–473 (2012).

59. R. P. Badman, F. Ye, W. Caravan, M. D. Wang, High trap stiffness microcylinders for nanophotonic trapping. *ACS Appl. Mater. Interfaces* **11**, 25074–25080 (2019).

60. B. C. Daniels, S. Forth, M. Y. Sheinin, M. D. Wang, J. P. Sethna, Discontinuities at the DNA supercoiling transition. *Phys. Rev. E* **80**, 040901 (2009).

61. P. Lebel, A. Basu, F. C. Oberstrass, E. M. Tretter, Z. Bryant, Gold rotor bead tracking for high-speed measurements of DNA twist, torque and extension. *Nat. Methods* **11**, 456–462 (2014).

62. A. Celedon, I. M. Nodelman, B. Wildt, R. Dewan, P. Searson, D. Wirtz, G. D. Bowman, S. X. Sun, Magnetic tweezers measurement of single molecule torque. *Nano Lett.* **9**, 1720–1725 (2009).

63. F. Mosconi, J. F. Allemand, V. Croquette, Soft magnetic tweezers: A proof of principle. *Rev. Sci. Instrum.* **82**, (2011), 034302.

64. J. Lipfert, J. W. J. Kerssemakers, T. Jager, N. H. Dekker, Magnetic torque tweezers: Measuring torsional stiffness in DNA and RecA-DNA filaments. *Nat. Methods* **7**, 977–980 (2010).

65. G. Volpe, G. Volpe, Simulation of a Brownian particle in an optical trap. *Am. J. Phys.* **81**, 224–230 (2013).

66. G. Ghosh, Dispersion-equation coefficients for the refractive index and birefringence of calcite and quartz crystals. *Opt. Commun.* **163**, 95–102 (1999).

67. J. F. Marko, E. D. Siggia, Stretching DNA. *Macromolecules* **28**, 8759–8770 (1995).

68. M. D. Wang, H. Yin, R. Landick, J. Gelles, S. M. Block, Stretching DNA with optical tweezers. *Biophys. J.* **72**, 1335–1346 (1997).
